# Epidemiological investigation of recurrent outbreaks of haemolytic uraemic syndrome caused by Shiga toxin-producing *Escherichia coli* serotype O55:H7 in England, 2014–2018

**DOI:** 10.1017/S0950268821000844

**Published:** 2021-04-19

**Authors:** C. Sawyer, B. Vishram, C. Jenkins, F. Jorgensen, L. Byrne, A. F. W. Mikhail, T. J. Dallman, K. Carroll, L. Ahyow, Q. Vahora, G. Godbole, S. Balasegaram

**Affiliations:** 1 Health Protection Division, Public Health Wales, Tyndall Street, Cardiff CF10 4BZ, UK; 2 National Infections Service, Public Health England, 61 Colindale Ave, London NW9 5HT, UK; 3 Public Health England Food Water and Environmental Microbiology Laboratory Porton, Salisbury SP4 0JG, UK; 4 PHE South East, Surrey and Sussex HPT, Parkside, Chart Way, Horsham RH12 1XA, UK; 5 East Midlands Health Protection Team, Public Health England, Nottingham NG2 4LA, UK; 6 Field Services, National Infection Service, Public Health England, London, UK

**Keywords:** Food-borne zoonoses, gastrointestinal infections, outbreaks, public health emerging infections, Shiga-like toxin-producing *E. coli*

## Abstract

Recurrent outbreaks of haemolytic uraemic syndrome (HUS) caused by Shiga toxin-producing *Escherichia coli* (STEC) serotype O55:H7 occurred in England between 2014 and 2018. We reviewed the epidemiological evidence to identify potential source(s) and transmission routes of the pathogen, and to assess the on-going risk to public health. Over the 5-year period, there were 43 confirmed and three probable cases of STEC O55:H7. The median age of cases was 4 years old (range 6 months to 69 years old) and over half of all cases were female (28/46, 61%). There were 36/46 (78.3%) symptomatic cases, and over half of all cases developed HUS (25/46, 54%), including two fatal cases. No common food or environmental exposures were identified, although the majority of cases lived in rural or semi-rural environments and reported contact with both wild and domestic animals. This investigation informed policy on the clinical and public health management of HUS caused by STEC other than serotype O157:H7 (non-O157 STEC) in England, including comprehensive testing of all household contacts and household pets and more widespread use of polymerase chain reaction assays for the rapid diagnosis of STEC-HUS.

## Introduction

Shiga toxin-producing *Escherichia coli* (STEC) are zoonotic, human gastrointestinal pathogens that cause symptoms ranging from mild, self-limiting diarrhoea to bloody diarrhoea [1]. However, more severe complications, including haemolytic uraemic syndrome (HUS) can occur [2]. STEC-related HUS, characterised by the presence of a triad of symptoms (thrombocytopenia, microangiopathic haemolytic anaemia and acute kidney injury) is the leading cause of acute renal failure in children under 10 years old and can result in life-long morbidity, and can be fatal [3, 4]. The animal reservoir for STEC is the gastrointestinal tract of ruminants, particularly cattle and sheep. These animals do not exhibit symptoms, but once colonised they can shed STEC for prolonged periods, characterised by fluctuating concentration of the organism in the faecal pat [5]. Transmission occurs via the faecal-oral route, with human infections occurring through direct contact with animals and their environments, or through contaminated food and water [1]. In household and institutional settings, secondary, person-to-person transmission of STEC has been described [6].

Whilst there are over 100 reported serogroups of STEC that can cause gastrointestinal disease in humans, in England the most commonly identified serotype is STEC O157:H7 [1, 7]. The virulence of STEC depends on the presence of Shiga toxins 1 and/or 2 *(stx*1 or *stx*2 genes) which can be subdivided into subtypes (*stx1a-1d* and *stx2a-2g).* There are certain subtypes, specifically, Stx2a and Stx2d, which, in conjunction with the presence of the *E. coli* attachment and effacing (*eae*) gene, are associated with an increased risk of STEC-HUS [8]. Whilst in England, STEC-HUS has historically been associated with STEC O157:H7 infections [1], there have been a number of outbreaks in Europe of non-O157 STEC which have resulted in high incidences of STEC-HUS, most notably, the STEC O104:H4 outbreak in Germany in 2011, which resulted in the largest outbreak of STEC-HUS recorded globally [9–12].

Understanding the underlying risk factors is the key to control the spread of STEC infection, and risk factors vary between outbreaks, and between different serotypes. While STEC O55:H7 infections are rare, series of recurrent outbreaks occurred in England between 2014 and 2018. A number of questions remain unanswered regarding the vector, source and routes of transmission of STEC O55:H7. In this paper, we review the epidemiological evidence collected over the 5-year period 2014–2018, to elucidate the plausible source(s) and routes of transmission of the causative agent, and to assess the on-going risk to public health.

## Methods

### Epidemiology

#### Case definitions

A confirmed case of STEC O55:H7 was defined as an individual with (i) a microbiologically confirmed isolate of STEC O55:H7 identified by culture from a faecal sample, and/or (ii) antibodies to the lipopolysaccharide (LPS) of *E. coli* O55 from serology, with a sample date in 2014–2018.

A probable case was defined as an individual with symptoms including diarrhoea, bloody diarrhoea or HUS, who had a known epidemiological link to a confirmed STEC O55:H7 case or an individual who had a positive polymerase chain reaction (PCR) test for *stx* genes and an epidemiological link to a confirmed STEC O55:H7 case.

Cases were classed as either symptomatic (individuals who reported experiencing diarrhoea, bloody diarrhoea or HUS) or asymptomatic. An outbreak of STEC O55:H7 was defined as a cluster of cases with isolates belonging to the same five single nucleotide polymorphism (5-SNP) single linkage cluster and epidemiologically linked on the basis of person, place and time.

#### Exposure histories

Questionnaires were administered to all suspected STEC cases by Public Health England (PHE), in line with standard operating procedures using enhanced surveillance questionnaires (ESQ) [1]. We collected demographic and clinical information from cases, as well as exposure information (food, travel, water, environmental and animal exposures) for the 14 days prior to the case's onset of illness – 7 days additional to the standard exposure period covered by the ESQs used routinely.

Additional questionnaires were developed to identify shared environmental exposures such as local sites, premises, environments or attractions visited by the cases. We developed these location specific questionnaires using information from cases' initial ESQs. Information provided by cases was added to an outbreak specific questionnaire for use prospectively. Cases who had already been identified and interviewed were re-interviewed and asked about any new areas identified by more recent cases.

#### Case–case study

A univariate case–case study was undertaken to compare clinical outcomes and symptoms of STEC O55:H7 cases to STEC O157:H7 cases with the same virulence profile as the STEC O55:H7 *stx2a/eae* outbreak cases. STEC O157:H7 *stx2a/eae* cases notified to PHE in the same period as the O55:H7 *stx2a/eae* cases with an ESQ available on NESSS, were included. We performed univariate logistic regression and calculated odds ratios (OR) and 95% confidence intervals (CI) using STATA 15.

### Microbiology

#### Microbiological detection and identification of cases

PHE STEC operational guidelines recommend referral of faecal specimens or rectal swabs from patients diagnosed with HUS, and/or bloody diarrhoea where STEC infection is suspected, and faecal specimens that test positive for *stx* by PCR at local hospital laboratories, to the Gastrointestinal Bacteria Reference Unit (GBRU) at PHE (STEC Operational Guidelines). At GBRU, faecal specimens are tested by PCR and cultured on MacConkey, Sorbitol MacConkey (SMAC) and cefixime-tellurite SMAC (CT-SMAC) agar [13]. For all positive specimens, 10 colonies were retested using the same PCR. Those colonies testing positive for *stx* were identified and sequenced. Serum samples were taken from patients with HUS when STEC was not detected in their faecal specimen by culture and were assessed for the presence of antibodies to the LPS of *E. coli* O26, O55, O103, O111, O145 and O157 [14].

All isolates of STEC O55:H7 from the human cases and two animal faecal specimens together with nine isolates of STEC O55:H7 from the PHE archive isolated between 2012 and 2014 from patients resident in the Republic of Ireland, were whole genome sequenced on the Illumina HiSeq platform, as previously described [15].

#### Data availability statement

FASTQ reads from all sequences in this study can be found at the PHE Pathogens BioProject at the National Center for Biotechnology Information (accession number: PRJNA315192).

### Environmental sampling

#### Food, water, environmental and animal samples

Environmental Health Officers (EHOs) from local authorities where cases were resident, inspected venues and sites that cases reported having visited in the 14 days prior to their onset and took appropriate samples. In the initial outbreak, this included food premises, however in subsequent outbreak investigations, the focus of sampling was environmental, following the hypothesis generated during the first outbreak that an environmental source was probable.

#### Dorset 2014–2015

During the initial outbreak investigation in Dorset, extensive food, water and environmental sampling was undertaken by EHOs, using standard samples and swabs, faecal pots and boot socks (a previously described method of sample collection originally conceived to detect *Campylobacter* present in the environment http://enigmaproject.org.uk/the-project/) [16]. Hydrological data were analysed, as was the movement of cattle and bird populations. Flood prone areas were targeted for sampling, with specific focus on areas with wildlife activity and those downstream of cattle populations, as identified by agricultural census data. Waterways and surface water runoff were sampled by the national Environment Agency Wessex office, Blandford, Dorset.

A positive cat faecal sample was identified in July 2014, and subsequently faecal specimens were collected from pet dogs and cats belonging to each case, where possible. Seventeen faecal specimens were taken from animals at a petting farm, and veterinary inspection and risk assessment of the premise. The Food Standards Agency carried out supply chain investigations linked to food consumed by cases, and their pets, focusing on identifying any retail distribution chains local to the Dorset area which differed to the rest of the country.

#### Surrey, 2016–2017

Following the hypothesis generated in the initial outbreak that the likely source of the outbreak was zoonotic, the sampling strategy during the Surrey outbreak focussed on environmental exposure and domestic and wild animal contacts. There were 30 samples taken in total, from livery stables, local recreation grounds and gardens of cases – all of which were identified as sites for environmental sampling through the process of iterative interviewing of cases.

#### East Midlands and east of England, 2018

Environmental samples were taken from play areas, multiple rural areas and woodlands which had all been visited by the cases in the 14 days prior to onset of illness. Boot sock sampling was undertaken in the areas most frequented by the cases and their contacts, including gardens, play parks and footpaths and surface swab samples were taken from garden bird feeders and poultry feeders. Outdoor toys, shoes and the wheels of a buggy were also swabbed with surface swabs. Food, drinking water, animal feed, soil and vegetation samples were also taken in addition to animal faecal samples which were collected from both wild and domestic animals from the area.

#### Food, water and environmental specimens

Animal faecal specimens were tested at GBRU using the same protocol as for human faecal specimens. All other samples were analysed by PHE's Food, Water and Environmental (FWE) Microbiology laboratories. At FWE, real-time PCR was used to examine samples for the presence of STEC based on CEN/ISO TS 13136 (https://www.iso.org/standard/53328.html). Water samples (up to 1 l) were filtered and filtrates enriched in 100 ml enrichment broth. Boot socks were immersed in 250 ml enrichment broth and swabs were immersed in 90 ml enrichment broth. The enrichment broths were then tested by PCR targeting *stx* described above (https://www.iso.org/standard/53328.html).

## Results

### Epidemiological investigations

**Fig. 1.**
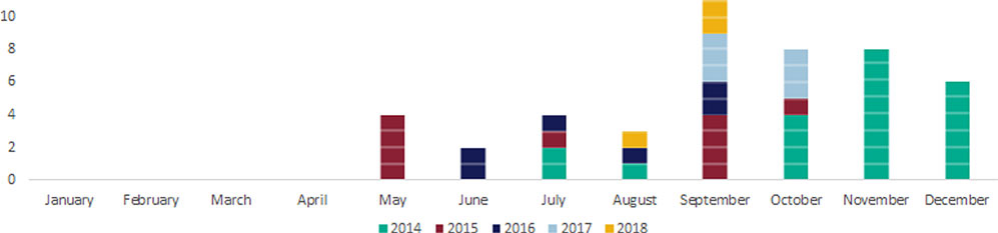
Epidemiological curve showing cases of STEC O55:H7 in England by month and year of onset, 2014–2018.

**Fig. 2.**
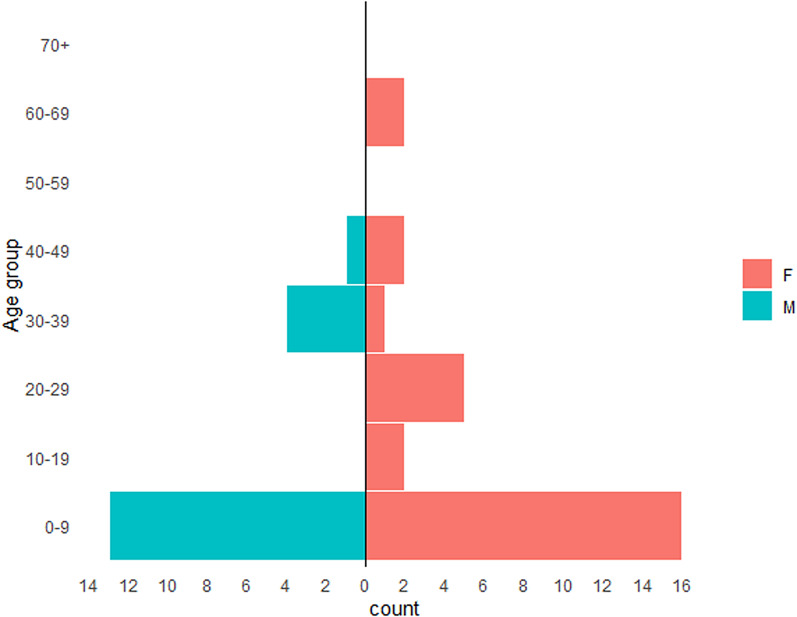
Age/sex pyramid of all cases of STEC O55:H7 in England, 2014–2018.

**Fig. 3.**
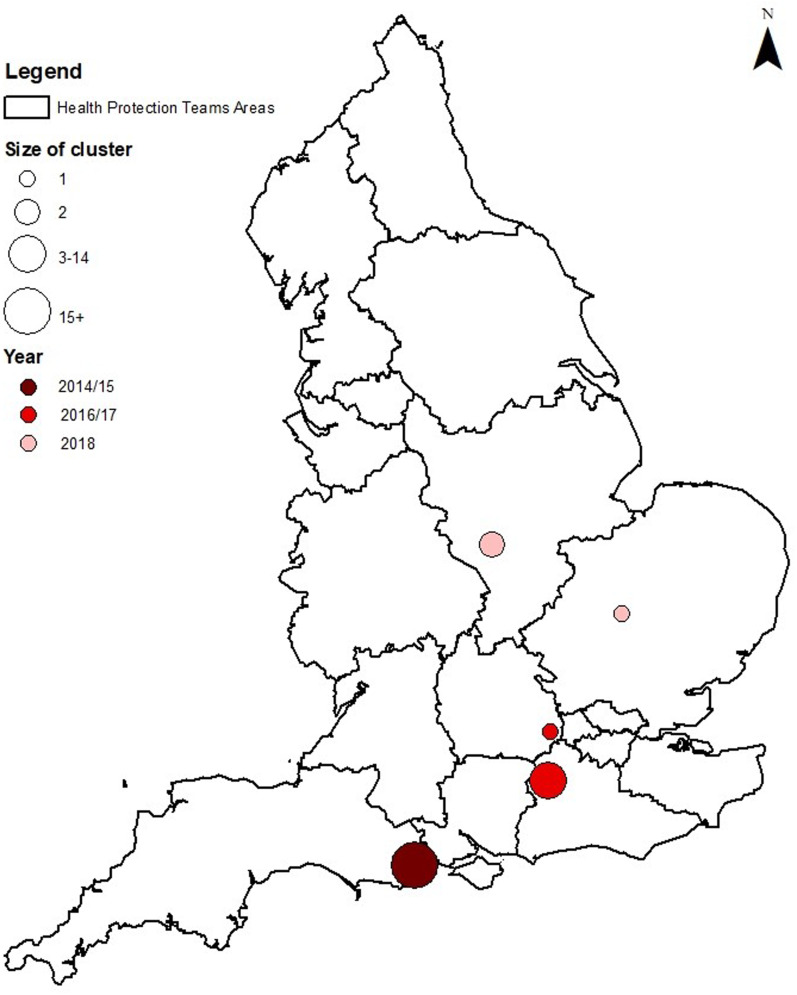
Map showing the geographical distribution of cases.

During the period 2014–2018, there were 43 confirmed and three probable cases of STEC O55:H7 identified (Table 1 and Fig. 1). The median age of all cases (confirmed and probable) across the period was 4 years old (range 6 months to 69 years old). The median age of the confirmed cases only was slightly lower, at 3 years old (range 6 months to 69 years) (Fig. 2). Over half of all cases were female (28/46, 61%) (Fig. 2). Thirty-eight cases were classed as being part of either one of two outbreaks of STEC O55:H7 (Dorset, 2014–2015 and Surrey, 2016–2017), three cases were classified as ‘sporadic’ and two cases were part of a distinct household cluster (Figs 1 and 3).

**Table 1.** Description of STEC O55:H7 cases, 2014–2018

Variable		Year					Total
		2014	2015	2016	2017	2018	
Total number		21	10	6	6	3	46
Case definition	Confirmed	21	10	6	3	3	43
	Probable	0	0	0	3	0	3
Symptoms	Symptomatic	13	9	6	5	3	36
	Asymptomatic	8	1	0	1	0	10
Epidemiological link to an outbreak	Sporadic	0	1	1	0	1	3
	Outbreaks cases	21	9	5	6	2	43
Age	Under 18	15	5	4	3	3	30
	Over 18	6	5	2	3	0	16
Sex	Male	10	4	2	1	1	18
	Female	11	6	4	5	2	28

In the Dorset outbreak, the median age of cases (*n* = 31) was 4 years old (range: 8 months to 69 years old) and in Surrey (*n* = 12), the median age was slightly older at 7 years old (range: 1–34 years old). The median age of the ‘sporadic’ cases (*n* = 3) was lower than the Dorset median, at 2 years old (range 6 months to 3 years old).

**Fig. 4.**
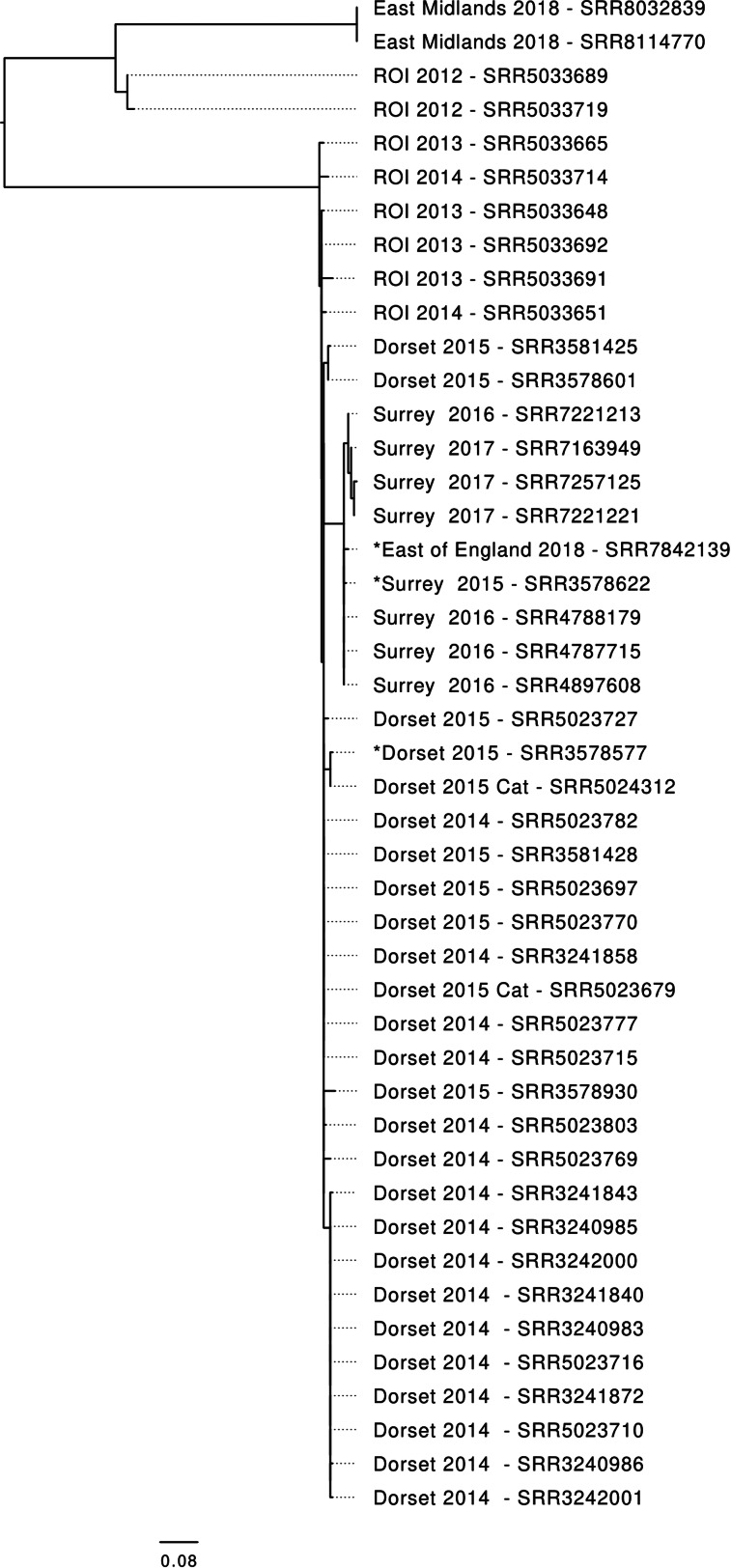
Phylogenetic tree showing the relationship between STEC O55:H7 linked to the recurrent outbreaks of in England between 2014 and 2018. All isolates were from human cases unless otherwise stated. Each sample is labelled by location, year of isolation and short read accession number. ROI – Republic of Ireland. *designated as a sporadic case.

Cases reported in the same year as one another were geographically clustered, within one or two postcode districts (Fig. 3). Postcode districts are areas in the UK which cover on average a population of around 23 000 and an area of 33 miles. In many instances, cases often lived within walking distance of one another. Three cases were not part of a spatio-temporal cluster (i.e. had no link to at least one other case by both place and time). However, although they appeared to be sporadic, analysis of the WGS data showed that these three cases were part of the same 5-SNP single linkage cluster (i.e. their isolates were less than 5-SNPs different from at least one other isolate in the cluster) as the Surrey outbreak (Fig. 4). One ‘sporadic’ case in 2016 was identified at the same time as the outbreak. This case was investigated as part of the outbreak following the analysis of the WGS data, despite not having a link to Surrey. Of the other two sporadic cases, one was identified in 2015 and the other in 2018, the years before and after the outbreak in Surrey (2016/2017). One had a link to Surrey, the other did not. With the exception of a person-to-person transmission event at a nursery school during the Dorset outbreak, and the cases belonging to the same household, none of the individuals linked to this outbreak had any contact with each other (Tables 2 and 3).

**Table 2.** Characteristics and clinical symptoms of STEC O55:H7 cases (confirmed and probable) by outbreak, 2014–2018

	Outbreak							
	Dorset (2014/2015)		Surrey (2016/2017)		Sporadic		All cases	
Median age	4 years old		7 years old		2 years old		4 years old
	*n*	%	*n*	%	*n*	%	
Female	17	54%	9	75%	2	67%	28	61%
HUS	15	48%	7	58%	3	100%	25	54%
Admitted to hospital	16	52%	8	67%	2	67%	26	57%
Bloody diarrhoea	15	48%	5	42%	3	100%	23	50%

**Table 3.** Odds of developing symptoms: STEC O55:H7 *Stx2a eae*  + ve compared to STEC O157:H7 *Stx2a eae*  + ve

Exposure	Odds ratio	95% CI	*P* value
HUS	33.4	14.65–76.08	*P* < 0.05
Death	11.18	0.78–156.12	*P* < 0.05
Hospitalisation	2.21	1.15–4.30	*P* < 0.05
Diarrhoea	0.39	0.17–0.93	*P* < 0.05
Bloody stools	0.58	0.30–1.12	0.079
Vomiting	0.91	0.45–1.77	0.761
Sex (female)	1.49	0.77–2.95	0.201

Onset of symptoms followed a seasonal pattern with onset of cases typically occurring between May and October for each year in which a case was reported (Fig. 1).

### Case finding

There were six cases identified in asymptomatic children during the screening of 99 children and staff from a nursery during the Dorset outbreak in 2014. In 2015, extended screening undertaken in Dorset of 4200 faecal specimens taken from bloody diarrhoea samples led to the identification of one additional case. Household contacts of STEC O55:H7 cases were screened for the infection, regardless of symptom history. Household screening of confirmed cases identified six further cases, of whom five (83.3%) were asymptomatic. This included household screening of families for two children with HUS, although STEC could not be isolated from their faecal specimens. The detection of STEC O55:H7 in the faecal specimens of household contacts provided the evidence for linking the two HUS cases to the STEC O55:H7 outbreaks.

### Clinical outcomes

Of the confirmed STEC O55:H7 cases (faecal culture positive, or with antibodies to O55:H7 LPS), 78.3% (36/46) were symptomatic (reported experiencing at least one of diarrhoea, bloody diarrhoea or HUS). The probable cases were symptomatic. Symptoms experienced by both confirmed and probable cases were severe – over half were hospitalised (56.5%) and developed HUS (54%), and half reported having bloody diarrhoea (50%). Two children aged 6 months and 2 years old, died as a result of the infection.

All symptomatic cases experienced diarrhoea, and of those who reported having diarrhoea, over two-thirds (64%) reported having blood in their diarrhoea.

In a univariate case–case analysis, STEC O55:H7 *stx2a/eae* cases were no more likely to be female than O157:H7 *stx2a/eae* cases (OR: 1.49, 95% CI: 0.77–2.95, *P* = 0.2), nor were they more likely to have bloody diarrhoea (OR: 0.58, 95% CI: 0.30–1.12, *P* = 0.09). However, STEC O55:H7 *stx2a/eae* cases were found to have over double the odds of being hospitalised compared to STEC O157:H7 *stx2a/eae* cases (OR: 2.2, 95% CI: 1.15–4.30, *P* < 0.05) and had over 30 times the odds of developing HUS (OR: 33.4, 95% CI: 14.65–76.08), *P* < 0.05) and over 10 times the odds of dying (OR: 11.2, 95% CI: 0.78–156.12, *P* < 0.05) compared to STEC O157:H7 *stx2a/eae* cases.

### Food histories, and animal and environmental exposures

Exposure questionnaires were available for 43 of the 46 confirmed or probable cases identified between 2014 and 2018. Questionnaires were unavailable for two confirmed cases notified in 2014 and for one probable case in 2017. Routine and enhanced questionnaires identified no common exposures between all cases. No common food and drink company, premises or supply chain was identified between cases nor was a common area/site or venue visited. The most frequently identified exposures were to rural/semi-rural environments and contact with animals (both domestic and wild). Several cases reported walking in local recreation grounds, woods, paddocks and in gardens (Table 4).

**Table 4.** Summary of exposures of cases of STEC O55:H7 from enhanced ESQs

Exposures of interest			Year					Total
			2014	2015	2016	2017	2018	
Total number of available ESQs			19	10	6	5	3	43
Visited environments		Farm/zoo	2	2	1	2	0	7
		Paddock	0	3	0	2	0	5
		Woods/fields	1	1	1	2	0	5
		Garden (soil)	0	1	0	2	0	3
		Beach (UK)	2	1	0	1	1	5
Animals	Any animal		7	7	3	5	3	25
	Domestic	Cats	4	5	1	3	1	14
		Dogs	5	4	2	3	3	17
	Wild	Rodents	0	0	0	0	0	0
		Birds	0	0	0	0	0	0
	Farm	Cattle (incl. calves)	0	0	0	0	0	0
		Sheep (incl. lambs)	1	0	0	0	0	1
		Horses	0	0	0	2	0	2
		Poultry	0	0	0	0	2	2
		Goats	1	1	0	0	0	2
Water	Swimming	Fresh water (including pools)	2	2	2	1	0	7
		Sea water	2	0	0	1	1	4
	Drinking	Private water source	0	0	0	0	0	0
		Mains	9	7	5	5	3	29
		Bottled	3	2	3	3	0	11

For the 43 cases for whom extended questionnaires were available, over half reported having contact with any animal (58%, 25/43), including dogs (68%, *n* = 17) and cats (44%, *n* = 14), horses (*n* = 2), goats (*n* = 2), poultry (*n* = 2) and sheep (*n* = 1). No cases reported contact with cattle or calves.

In total, six individuals reported that the animal that they had contact with had experienced diarrhoea in the 2 weeks before the onset of illness of the individual. Of the 17 cases who reported having contact with dogs, four reported that those dogs had a recent history of diarrhoea. One of the 14 cases who reported contact with cats reported that the cat had diarrhoea in the 2 weeks before the case's onset.

All but one case lived in rural or semi-rural environments. Cases reported day trips, including to, farms/zoos (*n* = 7), paddocks (*n* = 5), woods/fields (*n* = 5) and beaches (*n* = 5). Of those who visited a zoo/farm, three reported handling animals, including rabbits, chickens and goats. None of the reported zoos or farm were visited by multiple cases. Two reported consuming food at such sites and all reported hand washing at these sites.

All cases reported drinking mains or bottled water. There were seven cases who reported swimming in fresh water, sites for swimming included local swimming pools and in the woods. An additional four cases reported swimming in the sea. Again, no common beaches, pools or swim locations were shared between cases outside of household contacts.

### Environmental investigations

During the Dorset investigation, over 100 food, water and environmental samples were taken, none of which yielded a positive result for STEC O55:H7. The only positive samples were identified from faecal samples from two cats – one was from a pet cat belonging to a confirmed case and the other was a cat faecal sample taken from outside the home of another confirmed case. These samples were taken 4.5 km and 2 months apart. The isolates from these cats underwent WGS and they were found to match human isolates at the 5-SNP level. During the outbreak in Surrey, boot sock samples and animal faecal samples, which were presumed to be of canine origin, taken from a recreation site identified in ESQs completed by a number of cases, and geographically close to others, tested negative for STEC by PCR. In the East Midlands, after the two fatal cases occurred, a total of 31 samples were examined for STEC using real-time PCR testing. No STEC was detected, including STEC O55:H7 in any of the environmental swabs/samples taken. None of the samples taken of food tested positive for STEC by PCR.

### Microbiological investigations

Of the 43 confirmed cases, STEC O55:H7 was detected in 35 cases, all isolates carried *stx2a* and *eae*, the combination of which is associated with an increased risk of developing HUS [8]. All the strains of STEC O55:H7 described in this study were sensitive to tellurite and failed to grow on CT-SMAC, the selective media which is used to detect STEC O157:H7 in local hospital laboratories. The STEC O55:H7 strain that caused the fatal cases (*n* = 2) in the East Midlands was a sorbitol fermenting strain, however, all the other isolates were non-sorbitol fermenting (*n* = 35) most likely due to a non-sense mutation in *srlA*, previously described by Schutz *et al*. [15]. All the non-sorbitol fermenting isolates had acquired the antimicrobial drug resistance determinants *aadA-1b* encoding resistance to streptomycin and *dfrA-1* encoding resistance to trimethoprim.

Whole genome sequencing results from 35 human STEC O55:H7 cases and two cats were available. Of these, the sequences of STEC isolated from 33 of the cases and the 2 cats, belonged to the same 10-SNP single linkage cluster (Fig. 4). This 10 SNP single linkage cluster was subdivided into two 5-SNP clusters – one geographically linked to Dorset and the other, for the most part, to Surrey (Fig. 4). There were 24 human cases, including 10 cases linked to nursery school, and two cat samples belonging to the ‘Dorset’ 5-SNP cluster. There were nine cases from Surrey, London and the east of England in the ‘Surrey’ 5-SNP cluster. Eight STEC O55:H7 in the GBRU archive were isolated between 2013 and 2014 from individuals resident in the Republic of Ireland isolated, and six of these belonged to the same 5-SNP single linkage cluster as the isolates linked to the Dorset cases, and clustering at the 10-SNP level with those isolates from the cases in Surrey [15] (Fig. 4).

The sequences from the two cases from East Midlands in 2018 fell within 5-SNPs of each other but were phylogenetically distinct from the 10 SNP cluster, being more than 100 SNPs different from the sequences from the previous cases.

### Control measures

General advice about hand and food hygiene as well as methods for environmental decontamination was provided to all cases and parents of cases (where cases were children). In concordance with the PHE Interim Operations Guidance for STEC, both verbal and written advice was provided to cases and/or their contacts. Following the Dorset outbreak of 2014–2015, all STEC O55:H7 cases were interviewed about exposures in a 14-day period prior to onset of illness rather than the standard 7-day history. Following the identification of a positive stool sample from a cat, more specific hygiene advice around hand washing after interactions with domestic animals and before preparing food was provided.

Extensive contract tracing, including microbiological screening of all close contacts and exclusion from the workplace or school, where necessary, was performed. In all outbreaks and incidents of STEC O55:H7, briefings were issued to primary and secondary care services, including microbiology laboratories, to raise awareness in the health community and increase case detection.

## Discussion

In this study, we reviewed and analysed epidemiological and microbiological data linked to cases of STEC O55:H7 detected in the UK between 2014 and 2018 to look for evidence for the source and transmission routes of this emerging pathogen, and to monitor the on-going risk to public health. The number of cases declined between 2016 and 2018, although the case demographics were similar to those cases in the 2014 to 2015 Dorset cluster with respect to age, sex and seasonality [17]. The main difference between the two clusters was the geographical location of the cases, with the majority of cases from 2016 to 2018 residing in the south east and east of England. The geographical clustering of the Dorset cases led to the hypothesis that there was a local environmental source [17]. However, no cases have been detected in Dorset since 2015, and the change in geographical location raised further questions as to the source and transmission routes of the strain. To date, no additional cases of STEC O55:H7 with the same *stx* profile have been detected in the UK since 2018. Outbreaks of STEC O55:H7 have not been previously described in the literature in any other country, although there is evidence to suggest that *E. coli* O55:H7 was the progenitor from which STEC O157 has evolved [18].

Despite the decline in case numbers over the years, clinical outcomes of cases reporting symptoms remained at the severe end of the spectrum, including two fatal cases in 2018. The case–case study highlighted the severity associated with STEC O55:H7 infection compared to infection with STEC O157:H7. As previously discussed, this strain of STEC O55:H7 had a highly pathogenic combination of virulence factors, specifically *stx2a* and *eae*, known to be significantly associated with the potential to cause HUS [8, 19]. However, the symptoms reported by patients with STEC O55:H7 were significantly more severe than those reported by cases infected with STEC O157:H7 (*P* < 0.05) with the same virulence profile. Other potential factors contributing to poor clinical outcome may include the age of the affected population (children under the age of 5 years old) [18], high infectious dose (direct contact with animal faeces compared to contaminated food) and/or delayed diagnosis. The higher hospitalisation rates associated with STEC O55:H7 compared to STEC O157:H7 may be due to a higher likelihood of detecting STEC O157:H7 in cases of mild illness because the method for detecting STEC O157:H7 are relatively simple and well established. Despite the majority of cases reporting severe symptoms, 10 cases were identified during contact tracing activities and were recorded as asymptomatic. It was difficult to say for certain whether or not these cases had symptoms but failed to report them, however, asymptomatic infection which other STEC serogroups has been described.

Although we were unable to pin-point the source of the aetiological agent, we analysed the genomic data to better understand the transmission dynamics of this pathogen. STEC O55:H7 had not been observed in England prior to 2014 [17]. Previously, we have described two mechanisms to explain how novel strains of STEC emerge [20]. The first is by acquisition of *stx* encoding phage (and therefore the ability to cause severe disease in the human population) in a strain of *E. coli* already endemic in cattle and sheep [21, 22]. The second is by the importation of a novel strain of STEC O157:H7, either via imported food, or by the migration of animals or people [23, 24]. In this scenario, the imported strain may, or may not, become endemic. During the initial investigations in Dorset in 2014 and 2015, all the cases were infected with isolates of STEC O55:H7 that were closely related and fell within a 5-SNP single linkage cluster. We hypothesised that the strain was imported from outside the UK, possibly by wild birds, with persistence in the local environment driven by small mammals and/or birds acting as transient vectors, with transmission to humans occurring via contact with the contaminated environment or via household pets [25–29]. Exhaustive food and environmental investigation failed to confirm or to disprove this hypothesis, and so it remains a plausible explanation for the emergence of this strain. The occurrence of the cases in the south east and east of England in 2016 and 2017 caused by a strain within a 10 SNP single linkage cluster of the Dorset strain, raised concerns that this strain may have colonised domestic cattle or sheep and become endemic. However, the absence of cases since 2018 indicates that this is unlikely. The strain responsible for the two cases in the East Midlands in 2018 was not closely related to the earlier strains (>100 SNPs different) and may have been caused by a separate importation event.

The highly pathogenic nature of this strain of STEC O55:H7 justified the in-depth investigations that followed, and we continue to monitor for signs of re-emergence. The investigations have informed policy on the clinical and public health management of HUS caused by non-O157 STEC in England. We found that sampling the environment was resource intense and the boot sock methodology has not been well validated or evaluated for the detection of STEC in the environment during an outbreak. We recommend caution regarding the use of the extensive resources required to carry out environmental sampling in this scenario. In contrast, comprehensive testing of all household contacts, regardless of whether or not they are symptomatic or in a risk group, is strongly recommended [30]. If food histories indicate that contaminated food is unlikely to be the vehicle, testing faecal specimens from household pets may identify the transmission vector. Most importantly, this investigation highlighted the need for more widespread use of rapid, diagnosis of STEC-HUS, and has been a driver in the move towards implementing commercial gastrointestinal PCR assays in local and regional hospital laboratories in England [31].
